# Evolutionary reconstruction of pattern formation in 98 *Dictyostelium* species reveals that cell-type specialization by lateral inhibition is a derived trait

**DOI:** 10.1186/2041-9139-5-34

**Published:** 2014-10-01

**Authors:** Christina Schilde, Anna Skiba, Pauline Schaap

**Affiliations:** College of Life Sciences, University of Dundee, Dundee, UK

**Keywords:** Evolution of multicellularity, lateral inhibition, cell sorting, position-dependent cell-type specification, evolutionary reconstruction, DIF-1 signalling, *Dictyostelium*

## Abstract

**Background:**

Multicellularity provides organisms with opportunities for cell-type specialization, but requires novel mechanisms to position correct proportions of different cell types throughout the organism. Dictyostelid social amoebas display an early form of multicellularity, where amoebas aggregate to form fruiting bodies, which contain only spores or up to four additional cell-types. These cell types will form the stalk and support structures for the stalk and spore head. Phylogenetic inference subdivides Dictyostelia into four major groups, with the model organism *D. discoideum* residing in group 4. In *D. discoideum* differentiation of its five cell types is dominated by lateral inhibition-type mechanisms that trigger scattered cell differentiation, with tissue patterns being formed by cell sorting.

**Results:**

To reconstruct the evolution of pattern formation in Dictyostelia, we used cell-type specific antibodies and promoter-reporter fusion constructs to investigate pattern formation in 98 species that represent all groupings. Our results indicate that in all early diverging Dictyostelia and most members of groups 1–3, cells differentiate into maximally two cell types, prestalk and prespore cells, with pattern formation being dominated by position-dependent transdifferentiation of prespore cells into prestalk cells. In clade 2A, prestalk and stalk cell differentiation are lost and the prespore cells construct an acellular stalk. Group 4 species set aside correct proportions of prestalk and prespore cells early in development, and differentiate into up to three more supporting cell types.

**Conclusions:**

Our experiments show that positional transdifferentiation is the ancestral mode of pattern formation in Dictyostelia. The early specification of a prestalk population equal to the number of stalk cells is a derived trait that emerged in group 4 and a few late diverging species in the other groups. Group 4 spore masses are larger than those of other groups and the differentiation of supporting cell types by lateral inhibition may have facilitated this increase in size. The signal DIF-1, which is secreted by prespore cells, triggers differentiation of supporting cell types. The synthesis and degradation of DIF-1 were shown to be restricted to group 4. This suggests that the emergence of DIF-1 signalling caused increased cell-type specialization in this group.

**Electronic supplementary material:**

The online version of this article (doi:10.1186/2041-9139-5-34) contains supplementary material, which is available to authorized users.

## Background

Multicellularity allows division of labour between cells and the construction of multi-layered tissues in which specialized cells perform different functions. Organs and their constituent tissues develop from undifferentiated cells in the early embryo in response to a succession of chemical stimuli. Such stimuli can already be present in the zygote, but are mostly produced during the course of embryogenesis. Depending on their rate of diffusion and half-life, these stimuli can affect differentiation over longer or shorter ranges in a position-dependent manner, with stimuli that are displayed on the cell surface acting only on neighbouring cells. Directional movement and selective adhesion of differentiated cells are additional important processes to shape the developing organism [1].

The Dictyostelid social amoebas display a simple form of multicellularity, where from seven to up to a million cells aggregate to form a fruiting body [2]. In the model *Dictyostelium discoideum*, the fruiting bodies consist of a mass of spores that is held aloft on a column of stalk cells, and three additional cell types that form a basal disc to support the stalk, and an upper and lower cup to support the spore mass, respectively. During *D. discoideum* development, prespore and prestalk cells differentiate in well-regulated proportions that reflect the ratio of spores and stalk cells in the fruiting body [3, 4]. Initially, the prestalk and prespore cells differentiate intermixed with each other. They next sort out by differential chemotaxis and cell adhesion to form anterior prestalk and posterior prespore tissues [5, 6]. The cells that will form the basal disc and lower and upper cup differentiate among the prespore cells, and then sort to either the anterior boundary of the prespore region, or to the rearguard [7, 8]. Polyketide based signals such as DIF-1 (Differentiation inducing factor 1), which are produced by prespore cells [9] cause the differentiation of these support cells [10].

All these studies have been focussed on a single species, *D. discoideum*. However, studies on other species, such as *Polysphondylium pallidum*, *D. lacteum* and *D. minutum* indicate that cell-type specification mainly occurs by positional transdifferentiation of prespore cells into stalk cells [11, 12]. These conflicting results have thus far not been placed into an evolutionary context. Molecular phylogenetic studies showed that the Dictyostelia can be subdivided into two branches each containing two major groups and some group-intermediate species, which may represent additional groupings [13–15]. *D. discoideum* is a member of group 4, a set that contains species which form robust fruiting bodies with large spore heads.

In this work we investigated patterns of cell differentiation in 98 species across all groupings. The results were mapped onto the molecular phylogeny in order to identify trends in the evolution of cell-type proportioning and pattern formation. Our results indicate that position-dependent transdifferentiation of prespore cells into stalk cells is the ancestral mechanism for cell-type specialization in Dictyostelia, with position-independent proportioning of prestalk and prespore cells and additional cell-type diversification occuring mainly in group 4.

## Methods

### Cell culture

Most species were grown in association with *Klebsiella aerogenes* on one fifth SM agar with 0.5% charcoal and some on one third LP with 0.5% charcoal [15]. Cells were harvested from growth plates, washed with phosphate buffer (PB) (10 mM Na/K-phosphate, pH 6.5) and distributed at 5 × 10^6^ to 3 × 10^7^ cells/cm^2^ on 2 × 2 cm squares of dialysis membrane (immunohistology) or nitrocellulose filters ((X-gal) β-galactosidase staining) supported by PB agar (1.5% agar in PB). Cells were incubated at the optimal development temperature for each species until the desired developmental stages were reached.

### Immunohistology

#### Preparation of prespore antibody

**Figure 1:**
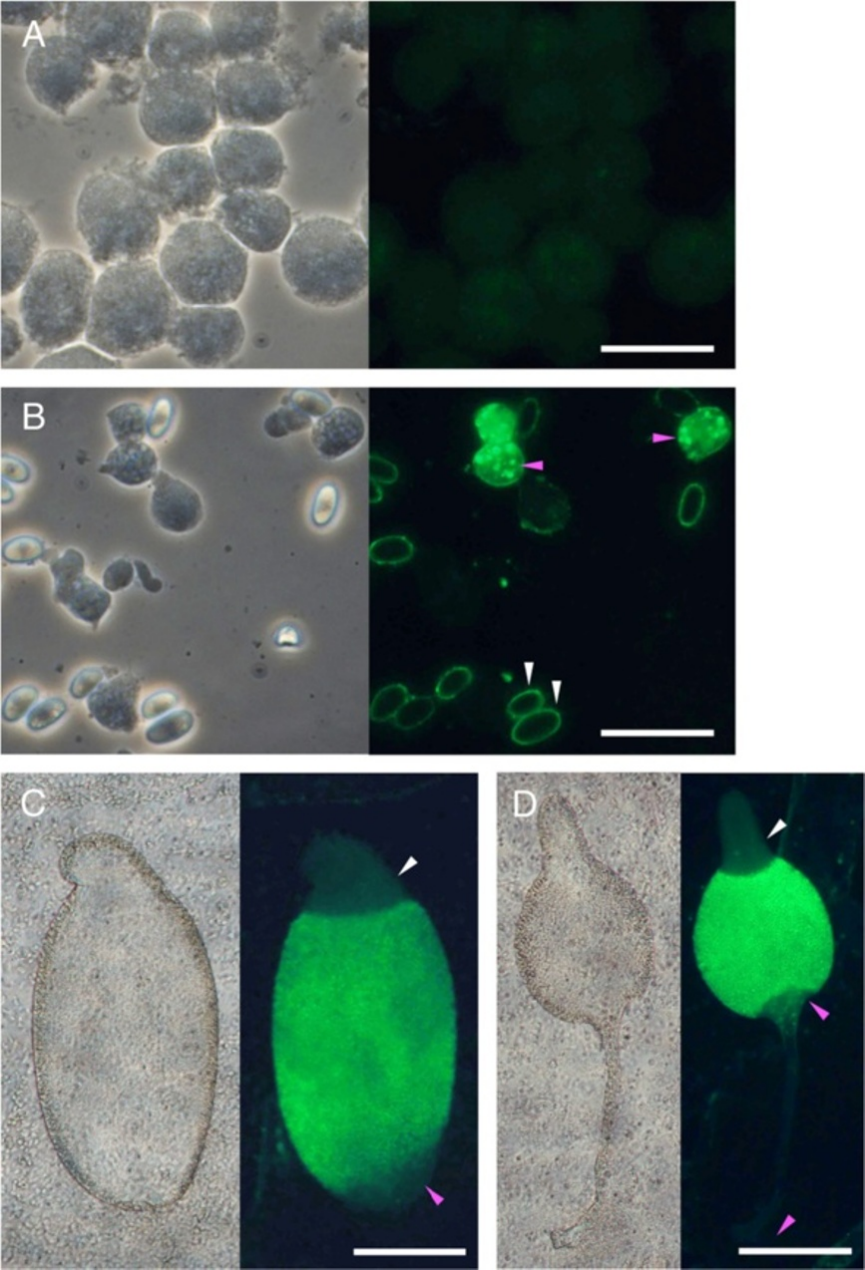
**Specificity of the prespore antibody.** Phase contrast and fluorescence images of vegetative cells **(A)**, cells from dissociated late culminants **(B)** and intact culminating **(C)** and mature fruiting bodies **(D)** of *D. discoideum*, which were stained with 1:2000 diluted antibodies, raised in rabbit against a mixture of *D. discoideum* and *P. pallidum* spores, and post stained with Fluorescein isothiocyanate (FITC)-conjugated donkey-anti-rabbit IgG. The spore antibodies react to spore walls (white arrows in **B**), and with vesicles in prespore cells, which presynthesize wall components (pink arrows in **B**). In multicellular structures stained prespore cells are separated from the unstained prestalk/upper cells (white arrows in **C**,**D**) by a well-defined boundary. The unstained rearguard cells (**C**, pink arrow) will form the lower cup and basal disc of the mature fruiting body (pink arrows in **D**). Scale bars, **A**/**B**: 10 μm; **C**/**D**: 100 μm.

*P. pallidum* PN500 and *D. discoideum* NC4 spores were harvested from fruiting bodies with 0.1% Triton in PB, sieved through mesh to remove stalks, washed several times in PB, and mixed with each other in a 1:1 ratio. Spore antibodies were raised in rabbits by Cambridge Research Biochemicals (Cambridge, UK). 1-ml aliquots of the final bleeds were incubated overnight with a 1-ml pellet of methanol-fixed *D. discoideum* and *P. pallidum* vegetative cells, mixed at a 1:1 ratio. After pelleting the cells by centrifugation for 30 minutes at 10.000 × g, the supernatant antibody fraction was stored as 10-μl aliquots at -80°C. Tests on several Dictyostelid species showed no reactivity of the adsorbed antibody to pre-aggregative cells, prestalk cells or stalks (Figure 1, Additional file 1: Figures A1-A3).

#### Immunostaining

Developed structures on the dialysis membrane were fixed for 15 minutes in ice-cold methanol. Structures were washed with PBS (0.8% NaCl in 10 mM Na/K phosphate, pH 7.4) with 5% BSA and incubated for 16 h at 4°C with a 1:2,000 dilution of pre-absorbed anti-prespore antibody in PBS/BSA. After three washes with PBS, structures were incubated with 1:100 diluted FITC-conjugated donkey-anti-rabbit-IgG for 2 h at room temperature. Structures were washed and mounted onto standard microscope slides for fluorescence microscopy and photography, using a Leica DMLB2 microscope and Qimaging Micropublisher 3.3 digital camera.

### Reporter gene constructs and transformation

The DNA sequences of all or most of the 5′intergenic regions of *P. pallidum* genes PPL_02670, PPL_04427, PPL_07208, PPL_07586, PPL_10235 and PPL_10763 were amplified from *P. pallidum* PN500 genomic DNA by PCR using the oligonucleotide primers listed in Table 1, yielding PCR products of 870, 4,475, 1,991, 2,687, and 1,077 bp respectively. In PPL_10763, an internal *Xba*I site (TCTAGA) was modified by mutagenesis into TCTCGA before cloning. The PCR products were directionally cloned into the restriction sites *Xba*I and *Bgl*II of vector pDdGal17 [16]. This vector expresses the *Escherichia coli* β-galactosidase (*LacZ*) gene under the control of the chosen promoters and contains the actin6-neomycin selection cassette. *P. pallidum* PN500 cells were transformed with the constructs as described previously [17] and selected for growth in the presence of 300 μg/ml G418.

**Table 1:** **Oligonucleotide primers used in this work**

Gene	Primer name	Restriction site	Sequence
PPL_02670	PPL_02670F	*Xba*I	5′-GA**TCTAGA**GTTTGTTGATATTCATATGTTTC-3′
PPL_02670	PPL_02670R	*Bgl*II	5′-CA**AGATCT**TTCCAGCATGAACCAATACAATTG-3′
PPL_10763	PPL_10763F	*Xba*I	5′-AGC**TCTAGA**CACTAACACACCACCACTTAATCACAC-3′
PPL_10763	PPL_10763R	*Bgl*II	5′-CAC**AGATCT**TGGTCGTAGTGGTGGTTGCTC-3′
PPL_10763	PPL_10763_mutXbaF		5′-AACTAACTTCTCGAAATAAACTATACT-3′
PPL_10763	PPL_10763_mutXbaR		5′-AGTATAGTTTATTTCGAGAAGTTAGTT-3′
PPL_07208	PPL_07208F	*Xba*I	5′-**TCTAGA**GGATGTATAATTATCTCATACTTCATCA-3′
PPL_07208	PPL_07208R	*Bgl*II	5′-**AGATCT**CGAAATGGCTTTGGTAATATTA-3′
PPL_07586	PPL_07586F	*Xba*I	5′-**TCTAGA**CCATTCGGATATCTAGTTTCCAAA-3′
PPL_07586	PPL_07586R	*Bgl*II	5′-**AGATCT**GCTGATAATATAATATTGAATTTCAT-3′
PPL_04427	PPL_04427F	*Xba*I	5′-**TCTAGA**ATAATCGAAATAAACAATATCAATA-3′
PPL_04427	PPL_04427R	*Bgl*II	5′-**AGATCT**AATATTATTTAAAAAAATATTAGTTATTTCTTTAA-3′

Restriction sites used for cloning are indicated in bold text.

### Visualization of β-galactosidase activity

Transformed cells were developed on nitrocellulose filters or dialysis membrane supported by PB agar until aggregates, primary sorogens and more mature fruiting structures with secondary sorogens had formed. Structures were fixed *in situ* with 0.25% glutaraldehyde and stained with 5-bromo-4-chloro-3-indolyl-β-D-galactopyranoside (X-gal) according to established procedures [18, 19]. Different developmental stages from cells transformed with the same constructs were stained for equally long periods, but for different constructs this could vary from 15 minutes to 24 h.

## Results

### Phylogeny-wide analysis of pattern formation

Cell differentiation patterns can be visualized by a range of techniques, such as *in situ* hybridization, analysis of cells transformed with fusion constructs of cell-type specific promoters and reporter genes, or with antibodies against cell-type specific proteins. However, the immense genetic diversity between the four major groups of Dictyostelia would necessitate the development of a vast number of probes, constructs or antibodies, which due to lack of gene or protein sequence information is not feasible.

Prespore cells in a number of species were previously shown to be specifically detected by antibodies raised against spores of a single species [11, 20]. We raised a universal antispore antibody by inoculating rabbits with a 1:1 mixture of spores from the group-4 species *D. discoideum* and the group-2 species *P. pallidum.* After pre-adsorbtion to fixed vegetative cells of both species, the antibody specifically recognised the vesicles in prespore cells, which partially presynthesize the spore wall of mature spores (Figure 1, Additional file 1: Figures A1-A3). In sorogens of *D. discoideum*, the spore antibody typically yields granular staining of prespore vesicles in the posterior 70% of the structure, leaving the most rearguard 5 to 10% and the anterior 20 to 25% free of granular staining. In these regions there is low homogeneous staining throughout the cells, which conveniently outlines the non-prespore tissue.

**Figure 2:**
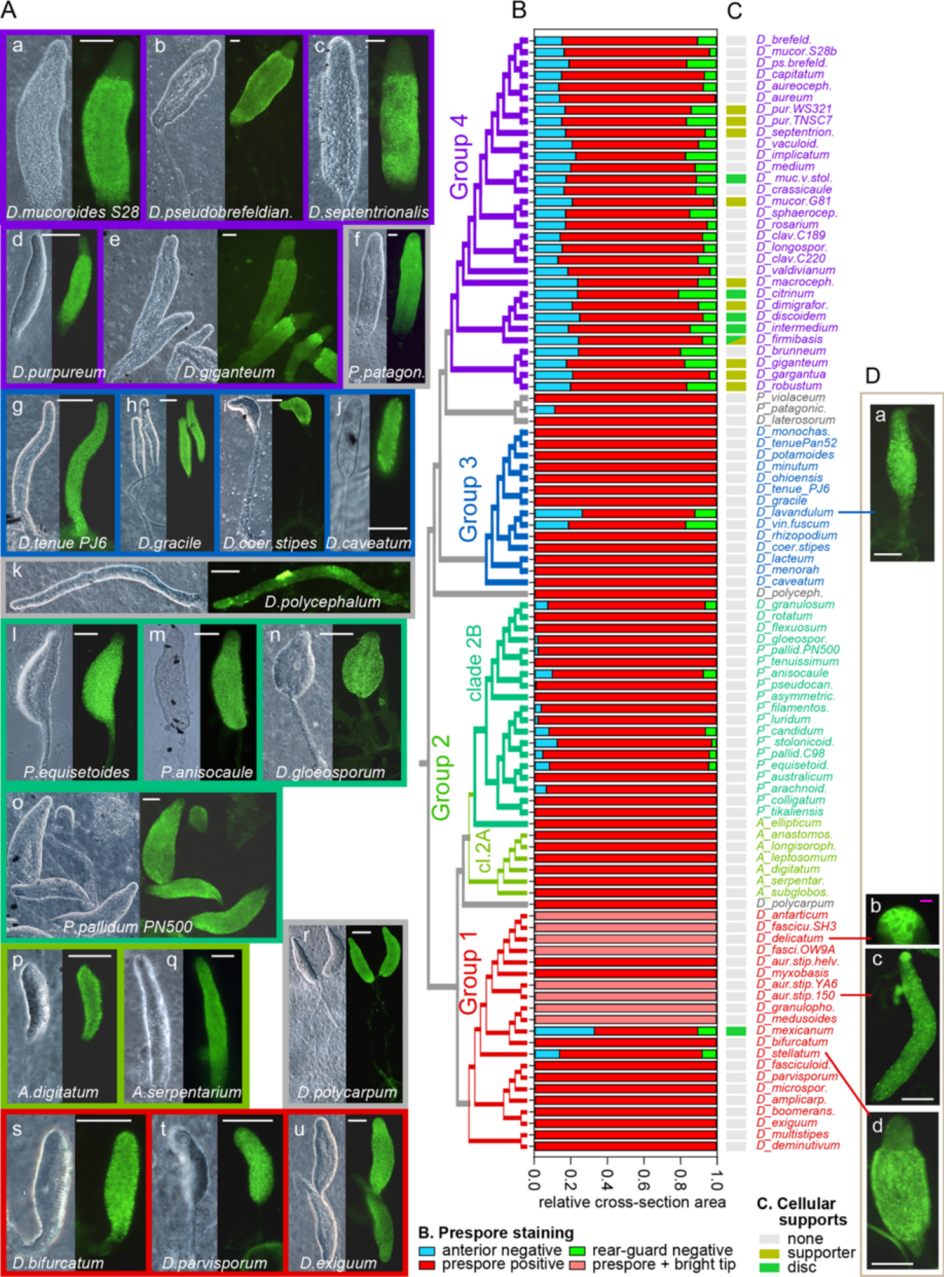
**Patterns of prespore differentiation across the Dictyostelid phylogeny. (A)** Cells from 98 *Dictyostelium* species that make up most of all major groups and intermediate minor groups of the *Dictyostelium* phylogeny were developed to the mid and late sorogen stage and stained with spore specific antibodies. Staining patterns for three to four representatives of each major group or clade and for the group intermediate species are shown together with phase contrast images of the sorogens. The coloured borders of the images mark the group or clade to which the species belong, as outlined in the cladogram of the *Dictyostelium* phylogeny [13, 15] in panel **B**. Scale bars: 100 μm. **(B)** The relative contributions of stained prespore and unstained anterior and rearguard cross-section areas were measured in five sorogens for each species and the averaged fractions of each area are presented as stacked bars, with red bars representing prespore area and blue and green bars anterior and rearguard non-prespore areas, respectively. **(C)** The presence or absence of cellular support structures, such as the basal disc or the triangular supporter were retrieved from a recent comparison of species phenotypes [15]. **(D)** Prespore staining patterns of a few species that were exceptions in their taxon group. White bars: 100 μm, pink bar 10 μm.

We used the spore antibody to identify prespore cells, defined by the presence of granules with spore antigens, in sorogens of 98 *Dictyostelium* species. Representative patterns in species from each of the four taxon groups and some group-intermediate species are shown in Figure 2A. The relative areas of tissues that reacted positively or negatively to the spore antibodies were measured by overlaying images of five stained sorogens for each species with a dot matrix and counting dots. These data are summarized with descriptive statistics and full species and strain names in Additional file 2: sheet 1, Prespore staining, and presented as stacked bar graphs in Figure 2B.

Similar to *D. discoideum*, all species in group 4 show a clearly defined pattern of unstained anterior cells, stained posterior cells, unstained rearguard cells and unstained stalks as exemplified in Figure 2A, a-e. In contrast, almost all species in group 3, except *D. lavandulum* and *D. vinaceo-fuscum* are stained along the entire length of the sorogen (Figure 2A, g-j). *D. lavandulum* and *D. vinaceo-fuscum*, which belong to a clade of four crampon-based species, show fairly large unstained anterior and rearguard regions (Figure 2B,Da). However, this is not the case for the two other members of the clade, *D. rhizopodium* and *D. coeruleo-stipes* (Figure 2A, i, and B).

All Acytostelids in clade 2A are stained along the entire length of the sorogen (Figure 2 panels B and A, p,q). However, many clade 2B species show a small unstained anterior region (Figure 2 panel B and A, l-o), and occasionally an unstained rearguard. In several species in the top clades of group 1, the spore antigen reacts particularly strongly to tip cells (Figure 2D, b,c), even after further pre-adsorption against their vegetative cells. However, while the staining distal from the tip is granular, suggesting that it is associated with prespore vesicles, the tip cells are stained at the periphery, suggesting that the antigen is present in the cell walls. Of this group of eight species, only *D. granulophorum* and *D. medusoides*, retain the peripheral spore antigen throughout the stalk (data not shown).

In the rest of group 1, the sorogens are stained along their entire length, but not in the stalks (Figure 2B,A, s-u). However, in two species, *D. mexicanum and D. stellatum*, substantial unstained anterior and rearguard regions are present (Figure 2 panels B and D, d). Most group-intermediate species are stained along their entire length (Figure 2A, f,k,r).

In *D. discoideum,* the non-prespore cells at the rearguard will form the basal disc and lower cup that support the base of the stalk and the spore head, respectively. The front-most non-prespore cells will form the stalk by reversing back through the centre of the cell mass, while the cells just anterior of the boundary with the prespore region will form an upper cup that caps the spore head. In the stalk and basal disc cells, autophagic vacuoles fuse to form a central large vacuole that takes up most of the cell volume [21] and the cells synthesize a cellulose-rich cell wall. The upper- and lower-cup cells remain amoeboid, but can be identified by the expression of specific marker genes [8]. Many species anchor their stalks to the substratum with buttresses of mucopolysaccharide matrix, but there are two other cellular supports, the crampon base, which is derived from tip cells and is essentially continuous with the stalk [22], and the triangular supporter, which is the equivalent of a basal disc for species that form a stalk during slug migration [23]. The presence or absence of a basal disc or supporter, as assessed previously [15], is plotted onto the phylogeny in Figure 2, column C. The species that have a basal disc or supporter also have an unstained rearguard region. However, the reverse is often not the case, although such species could have a lower cup, which is not readily recognizable by standard light microscopy.

### Expression domains of putative orthologues of *ecmA*and *ecmB*genes in *P. pallidum*

**Figure 3:**
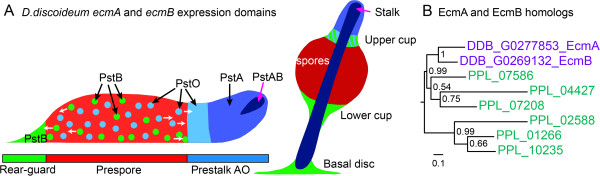
***ecmA*** **and** ***ecmB*** **expression domains and** ***P*** **.** ***pallidum*** **homologues. (A)** Expression domains of subregions of the *D. discoideum ecmA* and *ecmB* promoters, with PstO and PstA denoting expression from the distal PstO and proximal PstA regions of the *ecmA* promoter, respectively, and PstB and PstAB denoting expression from the distal basal disc, upper and lower cup region and the proximal stalk region of the *ecmB* promoter, respectively. The PstB cells sort to both the upper cup and the lower cup and basal disc of the fruiting body, while the PstO cells sort to the upper cup [8, 24, 25]. The cells that express *ecmA* and/or *ecmB* in the prespore region are collectively called anterior-like cells. **(B)** Protein sequences of the six top hits for a BLASTp search of the *P. pallidum* genome with *D. discoideum* EcmA and EcmB sequences were aligned with EcmA and EcmB, using Clustal Omega with five combined iterations [26]. The alignment was subjected to Bayesian phylogenetic inference until convergence had been reached, using a mixed amino acid model with a proportion of invariable sites [27]. The resulting tree is rooted at the midpoint between the most divergent sequences and shows the posterior probabilities of the interior nodes. The bar represents 0.1 substitutions per site.

The studies with the spore antibodies suggest that most taxa in groups 1 to 3 have prespore vesicles in all cells and therefore seemingly lack prestalk and rearguard cells. However, this can only be concluded from the absence of prestalk and rearguard cell markers. *D. discoideum* cells express two canonical prestalk genes, *ecmA* and *ecmB*, from complex promoters in subsets of prestalk and rearguard cells, which were designated pstO, pstA, pstB and pstAB cells by Williams and coworkers [8, 24]. These prestalk subtypes and their final positions in the fruiting body are summarized in Figure 3A. The pstO and pstA cell types were identified by expression of the *LacZ* reporter from distal and proximal regions of the *ecmA* promoter, respectively, and the pstB and pstAB cells, from distal basal disc, upper and lower cup and proximal stalk regions of the *ecmB* promoter, respectively.

Genomes of representative species of each taxon group were recently sequenced [28] (http://sacgb.fli-leibniz.de), but only *P. pallidum* from group 2 can thus far be genetically transformed. To assess the expression domains of prestalk genes in *P. pallidum*, we searched for ortholo24-gues of the *D. discoideum ecmA* and *ecmB* genes in the *P. pallidum* genome. Because both genes are members of a larger family of proteins that consist mostly of amino-acid cysteine-rich repeats, identification of the orthologues is not trivial. For both *ecmA* and *ecmB,* the gene with highest BLASTp local alignment score is *P. pallidum* gene PPL_07208, followed by genes PPL_07586, PPL_01266, PPL_10235, PPL_02588 and PPL_04427. Reverse BLASTp of PPL_07208 to all *D. discoideum* proteins identifies EcmA as the top hit, closely followed by EcmB. The other *P. pallidum* sequences either have EcmA, EcmB or DDB_G0279219 as first-reverse hits (Additional file 2: sheet 2). Similar to EcmA and EcmB, all encoded proteins consist of a signal peptide followed by highly similar cysteine-rich repeats.

To resolve orthology between the *D. discoideum* and *P. pallidum* proteins, a phylogenetic tree was constructed by Bayesian inference [27] from the fully aligned protein sequences (Figure 3B). With high statistical support, the tree shows that EcmA and EcmB form a separate clade, indicating that they emerged from a recent gene duplication. This clade groups together with PPL_07586, PPL_07208, PPL_04427, while PPL_01266, PPL_10235 and PPL_02588 form a separate grouping. High-throughput sequencing of mRNAs isolated during *P. pallidum* development (unpublished results P Schaap and G Gloeckner) showed that genes PPL_01266, PPL_10235, PPL_02588 are very poorly expressed during multicellular development, while genes PPL_07586, PPL_07208 and PPL_04427 show moderate to high expression levels at 14 to 32 h of development, when fruiting bodies are being formed (Additional file 2: sheet 3).

The complete 5′ intergenic regions of PPL_04427 and PPL_07586 and a 2-kb region upstream of the PPL_07208 start codon were fused to the *LacZ* reporter gene in pDd17 [16] and transformed into *P. pallidum* cells. All regulatory sequences in the *D. discoideum ecmA* and *ecmB* promoters are contained within a 1.6-kb region, upstream of the start ATG. The promoters were also fused to ala-gal and ile-gal reporter genes that generate stable and labile β-galactosidase proteins in *D. discoideum*, respectively [29]. However, in *P. pallidum*, such constructs did not yield any detectable β-galactosidase expression (data not shown).

**Figure 4:**
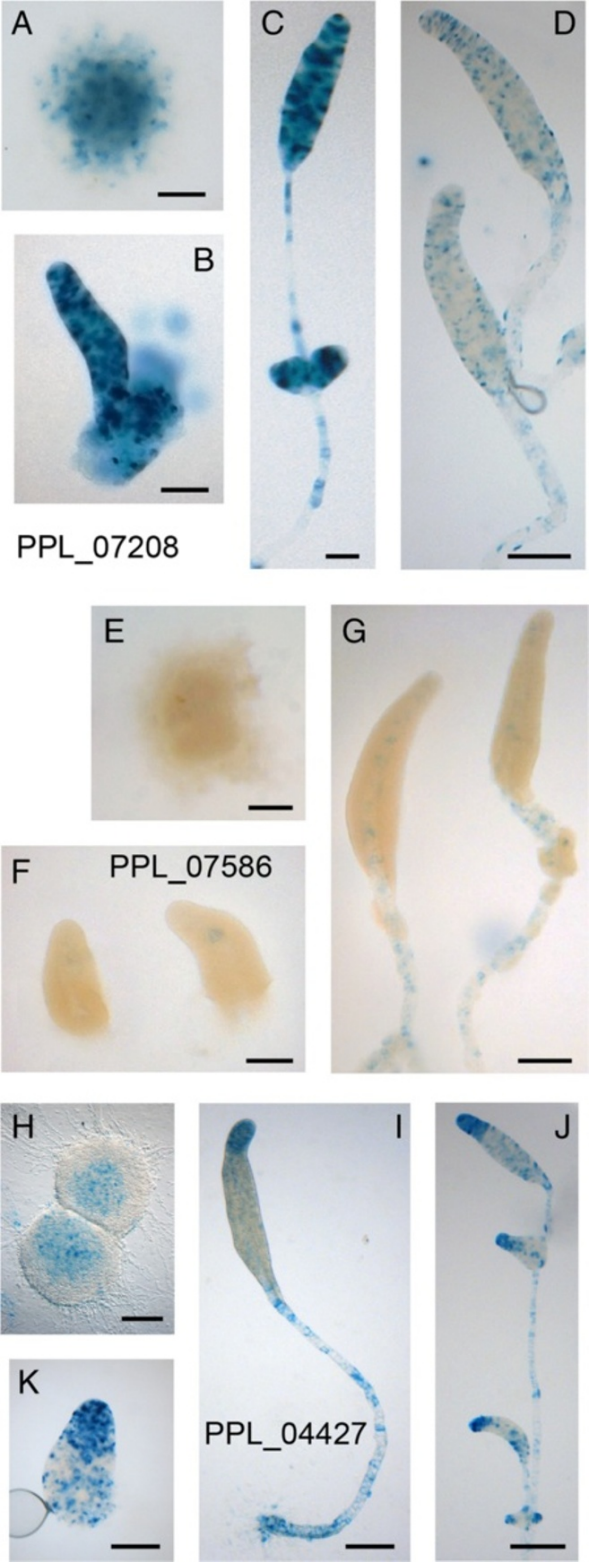
**Expression patterns of** ***P*** **.** ***pallidum ecmA*** **and** ***ecmB*** **homologues.** *P. pallidum* cells, transformed with fusion constructs of *LacZ* and the promoters of the closest *P. pallidum ecmA/ecmB* homologues PPL_07208 **(A-D)**, PPL_07586 **(E-G)** and PPL_04427 **(H-K)**, were developed to completed aggregates **(A, E, H)**, tipped mounds/early sorogens **(B, F, K)** and more mature sorogens **(C, D, G, I, J)** and then fixed and stained with X-gal. PPL_07208::LacZ structures stained very strongly within 1 hr **(A-C)** and were also stained more briefly **(D)**. PPL_07586::LacZ stained weakly after 24 h and PPL_04427::LacZ required 15 min for staining to develop. Bar: 100 μm.

The transformed cells were distributed on nitrocellulose filters or dialysis membrane supported by non-nutrient agar and developing structures were fixed and stained with X-gal. PPL_07208::LacZ expression was first detectable throughout the newly formed aggregate (Figure 4A), and remained localized throughout the entire primary sorogen, stalk and secondary sorogens (Figure 4B,C). When structures were stained briefly, the strongest staining cells were scattered among less stained cells (Figure 4D). Staining was only occasionally enriched at the sorogen tip. PPL_07586::LacZ was expressed only weakly in the inner tip of the sorogen and in the stalk. PPL_04427::LacZ was first expressed in the centre of aggregates. The emerging primary and secondary sorogens showed strongest expression at their anterior 15 to 20% and in the stalk, but there was also scattered expression throughout the sorogens. We conclude that the expression patterns of the *P. pallidum ecmA* or *ecmB* homologues only partially resemble those of *D. discoideum ecmA* and *ecmB*. PPL_07208 shows almost no cell-type specificity, while PPL_07586 and PPL_04427 are specifically expressed in the stalk, with PPL_04427 also being expressed in the anterior tip region. There is however no expression from either gene that would outline upper and lower cup or basal disc regions.

### Expression of *P. pallidum*orthologues of *D. discoideum*markers for prestalk subtypes

*In-situ* hybridization studies of *D. discoideum* expressed sequence tags (ESTs) identified a larger set of genes that are selectively expressed in prestalk cells in *D. discoideum*[30, 31]*.* We searched for orthologues of these genes in the *P. pallidum* genome by bidirectional BLASTp search and identified two genes that each detected their *D. discoideum* query sequence as the top scoring hit (Additional file 2: sheet 2). These genes also showed good expression during late development (Additional file 2: sheet 3).

**Figure 5:**
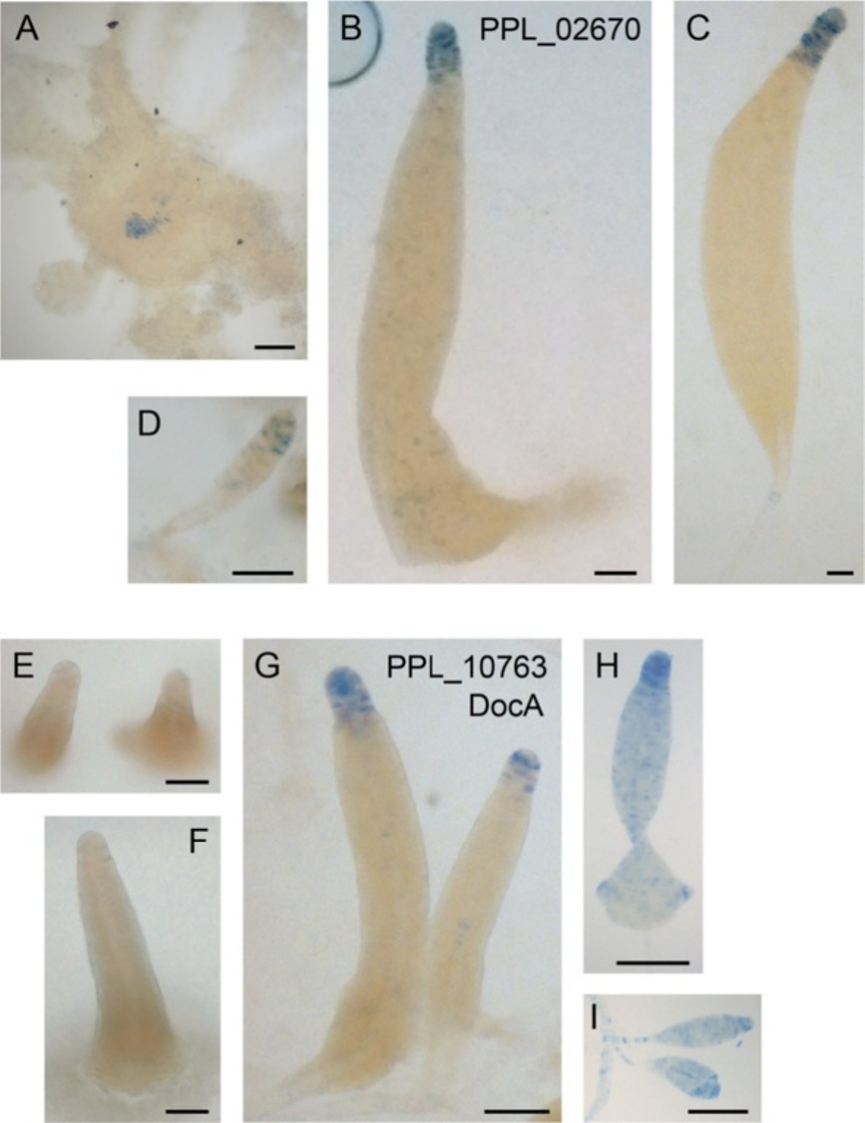
**Expression patterns of** ***P. pallidum*** **orthologues of** ***D. discoideum*** **PstOA and PstO genes.** *P. pallidum* cells transformed with *LacZ* fused to promoters of the *P. pallidum* orthologue PPL_02670 of *D. discoideum* pstO cDNA SSH630, and orthologue PPL_10763 of *D. discoideum* pstAO cDNA SLH511 [30, 31] were developed into aggregates **(A)** and different stages of primary **(B, C, E, F,G)** and secondary **(D, H, I)** sorogen formation, and stained with X-gal. Bar: 100 μm.

The *P. pallidum* orthologue PPL_02670 of the *D. discoideum* pstO gene DDB_G0272420 (Table 2) is first expressed in the centre of the aggregate that will form the tip (Figure 5A). Expression remains confined to the utmost tip in primary sorogens (Figure 5B,C), but becomes more scattered in secondary sorogens (Figure 5D). This expression pattern is quite distinct from its *D. discoideum* orthologue, which is not expressed at the tip, but in the upper cup region below the tip and more scattered lower down in the sorogen [30].

**Table 2:** ***P*** **.** ***pallidum*** **homologues and orthologues of** ***D*** **.** ***discoideum*** **prestalk genes**

***D*** . ***discoideum*** gene	Expressed sequence tag	Subtype	Expression domain		***P. pallidum*** gene
			Migrating sorogen	Culminating fruiting body	
DDB_G0277853 *EcmA*		pstAO	pstO + PstA	Tip + stalk + upper and lower cup	PPL_07208 PPL_07586 PPL_04427
DDB_G0269132 *EcmB*		pstAB	PstAB + PstB	Stalk + upper and lower cup + basal disc	
DDB_G0272420	SSH630	pstO	pstO	Upper cup	PPL_02670
DDB_G0291974 *DocA*	SLH511	pstAO	pstO + PstA	Tip + stalk + upper and lower cup	PPL_10763

*P. pallidum* orthologues of the canonical *D. discoideum* prestalk genes *EcmA* and *EcmB*[8] and of ESTs SSH630 and SLH511, which are specifically expressed in the pstO and PstAO prestalk subpopulations of *D. discoideum*[30], were identified using the criterion of best birectional hits in BLASTp queries of all protein sequences in either genome [28, 32]. The expression domains of the *D. discoideum* genes in sorogens and emerging fruiting bodies, as schematically represented in Figure 3, are summarized. *D. discoideum EcmA* and *EcmB* emerged from a gene duplication that occurred after groups 2 and 4 diverged (Figure 3B). They consequently have no single orthologues in *P. pallidum*, but a set of three homologues which show similar temporal expression as *EcmA* and *EcmB,* as determined by high-throughput RNA sequencing [33] (P Schaap and G Gloeckner, unpublished results). SSH630 and SLH511 are orthologous to PPL_02670 and PPL_10763, respectively. See Additional file 2: sheets 2 and 3 for BLASTp scores of bidirectional hits and temporal expression levels of the *D. discoideum* and *P. pallidum* genes, respectively.

The *P. pallidum* orthologue PPL_10763 of the *D. discoideum* pstAO gene *DocA* (Table 2) is first expressed at the tip of fully formed sorogens (Figure 5 E-G). At later stages PPL_10763 is also expressed in scattered cells throughout the primary and secondary sorogens and in the stalk (Figure 5H,I). However, expression remains strongest at the utmost tip. Also this pattern is different from its *D. discoideum* orthologue *DocA*, and from pstAO genes in general. Firstly, the *D. discoideum* genes are already expressed in the aggregate, and secondly they acquire specific expression domains at the base of the culminating fruiting structure and below the tip, that are not displayed by PPL_10763.

In conclusion, the expression patterns of PPL_10763, PPL_02670 and the ecmA/B homologue PPL_04427 show that despite the presence of prespore markers throughout almost the entire sorogen, *P. pallidum* does express prestalk genes at the anterior 10 to 20% of the sorogen. However, there is no evidence for the existence of upper and lower cup/basal disc expression domains.

## Discussion

### Most Dictyostelids form the stalk by positional transdifferentation of prespore cells

Visualization of spore antigen with universal *Dictyostelium* spore antibodies revealed that only the group-4 sorogens consistently show a clear demarcation between posterior prespore and anterior non-prespore cells. With only a few exceptions the species in the other groups express spore antigens along the entire length of the sorogen and only lose the antigen upon de-differentiation into stalk cells at the sorogen tip (Figure 2). However, in the group-2 species *P. pallidum,* orthologues and homologues of several *D. discoideum* prestalk markers do show expression at the anterior tip of the sorogens (Figures 4 and 5) in a region that overlaps with the expression of spore antigen. This could mean that either prestalk and prespore cells are intermixed at the anterior or that both prestalk and prespore markers are expressed by the same cells.

For several reasons we believe the latter to be the case. Transmission electron microscopy studies of the group-3 species *D. minutum* and *D. lacteum* and the group intermediate species *P. violaceum* showed that although the prespore vesicles, which prefabricate the inner layer of the spore wall, are present throughout the sorogen, their inner wall is disintegrating close to the sorogen tip and the vesicles are transforming into autophagic vacuoles, while the cells are entering the stalk [11, 34]. In *P. pallidum*, mRNA of Sp45, an homologue of the *D. discoideum* spore coat gene *CotA*, is not expressed in the anterior 15 to 20% of the sorogen [35], a region that is roughly equal to that of the anteriorly expressed genes PPL_04427, PPL_02670 and PPL_10763. Combined, these observations indicate that as cells are approaching the tip, where cells are continuously turning over into stalk cells, they stop expressing spore genes and start expressing stalk genes. However, it takes a while longer for all the spore antigen to be degraded.

The Acytostelid *A. subglobosum* shows an interesting variant of this process. Acytostelids, which reside in clade 2A, produce a central cellulose stalk tube, but do not incorporate stalk cells in this tube. Here, initially almost all cells express the spore gene *CotD* except those at mound tip, which express the prestalk gene *ecmA*. During sorogen formation, the *ecmA* expression domain expands downward at the expense of the *CotD* domain. However, the cells do not lose spore antigen or prespore vesicles and eventually all turn into spores. The *ecmA* mRNA becomes localized at the region of the (prespore) cell that faces the stalk tube and the cysteine-rich extracellular matrix protein that is encoded by *ecmA* probably acts to reinforce the stalk [36].

### Pattern formation in group 4 is dominated by lateral inhibition and cell sorting

Positional transdifferentiation is much less obvious in group-4 species. Disintegration of prespore vesicles across the prespore/prestalk boundary was observed in migrating sorogens of *D. mucoroides*[37] and *D. purpureum*[11], two group-4 species that, unlike *D. discoideum,* form a stalk during migration and thereby continually deplete the prestalk cells. Late culminants of *D. discoideum* also show some disintegration of prespore vesicles [38]. However, in general all group-4 species set aside a large proportion of prestalk cells at the onset of sorogen formation, thus reducing the need for transdifferentiation. It has been long debated whether this early prestalk differentiation is under positional control [39–41], or occurs scattered throughout the mound, followed by preferential sorting of prestalk cells towards the anterior tip [42–44].

The local appearance of differentiation markers can misleadingly indicate a positional signal, when actually cell sorting has occurred faster than differentiation becoming overt. The observation that prestalk and prespore cells differentiate intermixed with each other in normal proportions, when cell movement is pharmacologically inhibited, supports a model in which scattered differentiation is followed by sorting [44]. Prespore differentiation occurs in response to secreted 3′5′-adenosine monophosphate (cAMP) throughout Dictyostelia [19, 45]. In *D. discoideum,* the newly differentiating prespore cells secrete differentiation inducing factor 1 (DIF-1) [9], which induces cells to differentiate into PstB cells [10]. Other DIF-like factors, such as DIF-6 and DIF-7 are thought to induce other prestalk sub-types [46, 47]. The prestalk cells express a DIF-1 inactivating dechlorinase [48], which acts to halt prestalk differentiation, once a certain proportion of prestalk and prespore cells has been reached [49]. Such a system, akin to lateral inhibition [50], can accurately regulate cell-type proportions, irrespective of the size of the organism. This is advantageous for organisms like Dictyostelia, where cell numbers in multicellular structures can vary over four orders of magnitude.

### Regulation of prestalk and prespore cell proportions is an evolutionary novelty

Can we exclude that position-independent cell differentiation occurs in groups 1 to 3? Of the three *P. pallidum* homologues of the canonical *D. discoideum* prestalk genes *ecmA* and *ecmB*, one, PPL_07208, shows no cell-type specificity. For another, PPL_07586, expression is only visible in the stalk, while the third, PPL_04427, appears to be strongly expressed at the anterior region and in the stalk, but also shows scattered staining throughout the prespore region in a similar pattern to *ecmA* in *D. discoideum*. Expression of PPL_02670, an orthologue of the *D. discoideum* pstO marker SSH630, seems strictly tip-specific in primary sorogens. Expression of PPL_10763, an orthologue of the PstAO marker DocA is also first tip-specific, but later extends throughout the primary and secondary sorogens. Although initial expression seems positional for PPL_07586, PPL_02670 and PPL_10763, we can also here not exclude that cells initially expressed these genes, while scattered throughout the aggregate and then rapidly moved to the tip. The difference with *D. discoideum* is that if this were the case, the initial population of prestalk cells is far too small to account for all subsequent stalk cell differentiation, and continuous transdifferentiation of prespore cells would be required. There is therefore no early proportioning of prestalk and prespore cell types in *P. pallidum.*

The expression pattern of PPL_04427 appears to indicate the presence of anterior-like cells in *P. pallidum* sorogens. There is however a marked difference in the fate of these cells compared to *D. discoideum*. In *D. discoideum* such cells will sort out to form clearly defined upper and lower cup/basal disc regions, while in *P. pallidum* these regions are absent. A fusion of the *D. discoideum ecmB* promoter with *LacZ* was previously introduced in *P. pallidum,* where it is only active in the tip and stalk cells of primary and secondary sorogens without showing the upper and lower cup staining that marks its expression in *D. discoideum*[12]*.* Conversely, *D. minutum* (group 3) *ecmB-LacZ* expressed in *D. discoideum* is only active in the stalk [51]*.* Apparently, no sequences responsible for upper and lower cup expression are present in *D. minutum ecmB*, and they are not recognized by *P. pallidum* in the *D. discoideum ecmB* promoter.

In *D. discoideum*, the lower cup and basal disc cells are induced by DIF-1 [10]. DIF-1 was also shown to be synthesized and dechlorinated by another group-4 species, *D. mucoroides. P. violaceum*, which is positioned between groups 3 and 4 can synthesize, but not dechlorinate DIF-1, while *D. minutum* and *D. vinaceo-fuscum* in group 3 cannot do either [52, 53]. DIF-1 synthesis was also found absent from the group-2 species *A. subglobosum*[54]. It is therefore very likely that DIF-1 and the cell-types that it induces only emerged late in the lineage leading to group 4.

A number of species throughout groups 1 to 3 show regions at the anterior and/or rearguard that do not react with spore antibodies. One species, *D. mexicanum* in group 1, even makes a cellular basal disc [55]. There is currently no genome information on these species and therefore we cannot assess to what extent they resemble the prestalk and rearguard regions in group 4. It is possible that these species, which are all late diverging within their taxon groups, have independently invented cell-type proportioning and novel cell-type specialization. All early diverging species and species intermediate to groups 2 and 3 form the stalk by positional transdifferentiation of prespore cells (Figure 2B) and this is therefore very likely the ancestral mode of pattern formation in the Dictyostelia.

### Cell-type proportioning and increased cell specialization are positively correlated with fruiting body size

The mapping of morphological features to the Dictyostelid phylogeny revealed that group-4 species stand out by forming larger fruiting structures, with sorus diameter particularly being significantly larger than in groups 1 to 3. Group-4 species all use cAMP as attractant for aggregation and have lost encystation of individual amoebas as an alternative survival strategy [15]. Species in the other groups use other attractants for aggregation, but use secreted cAMP for organisation of cell movement during sorogen formation and for induction of prespore differentiation [15, 19, 56, 57]. While a causal relationship between early cAMP signalling, loss of encystation and increased cell-type diversity and proportioning is not immediately evident, it is plausible that the emergence of larger fruiting bodies in group 4 was dependent on the generation of specialized cell types to anchor the stalk and to support the spore head. Early cell-type proportioning, provides an additional advantage in not wasting resources in synthesis of spore wall components, only to degrade them later. The emergence of DIF signalling in group 4 may have been instrumental to those innovations.

## Conclusions

Systematic analysis of pattern formation accross the Dictyostelid phylogeny showed that all early diverging Dictyostelia and most species in taxon groups 1 to 3 first differentiate all cells in their sorogens into prespore cells. The stalk is formed by local transdifferentiation of prespore cells into stalk cells at the sorogen tip.

Species in group 4 set aside a large proportion of non-prespore cells at the onset of sorogen formation, which sort towards both the anterior and utmost rear of the sorogen to yield reservoirs of cells for differentiation into stalk cells and other somatic cell-types.

There is good evidence that this early non-positional cell-type proportioning is the result of factors secreted by prespore cells, and that the ability to synthesize these factors only emerged in the group-4 lineage.

## Electronic supplementary material

Additional file 1: **Specificity of antispore antibodies tested on three group-representative Dictyostelia.** This file contains **Figures** **A1-A3** that show staining of *D. lacteum*, *P. pallidum* and *D. fasciculatum* cells of different developmental stages with antispore antibodies. (PDF 4 MB)

Additional file 2: **Pattern quantitation, prestalk gene discovery and transcriptome data.** This spreadsheet contains a quantitation of prespore staining patterns in sheet 1, results of BLASTp searches for prestalk genes in *P. pallidum* in sheet 2, and transcriptomic data on the developmental regulation of prestalk genes in *P. pallidum* and *D. discoideum* in sheet 3. (XLSX 72 KB)

## Abbreviations

bp: base pairs: BLAST: basic local alignment search tool
BSA: bovine serum albumin
cAMP: 3′5′-adenosine monophosphate
CotA and CotD: Sporecoat proteins A and D
DIF-1: Differentiation Inducing Factor 1
DocA: Dedicator of cytokinesis A
EcmA and EcmB: Extracellular matrix proteins A and B
EST: expressed sequence tag
FITC: Fluorescein isothiocyanate
*LacZ*: β-galactosidase gene
PB: phosphate buffer
PBS: phosphate-buffered saline
Pst: prestalk
X-gal: 5-bromo-4-chloro-3-indolyl-β-D-galactopyranoside.
