# Supplementary material for: Evolutionary reconstruction of pattern formation in 98 Dictyostelium species reveals that cell-type specialization by lateral inhibition is a derived trait
Source: EvoDevo. 2014 Oct 1;5:34. doi: 10.1186/2041-9139-5-34 (PMC4406040; doi:10.1186/2041-9139-5-34)
Supplement: Supplementary file 1 — Additional file 1: Specificity of antispore antibodies tested on three group-representative Dictyostelia. This file contains Figures A1-A3 that show staining of D. lacteum, P. pallidum and D. fasciculatum cells of different developmental stages with antispore antibodies. (PDF 4 MB) [file 13227_2014_127_MOESM1_ESM.pdf]

Specificity of anti-spore antibodies tested on three group-representative Dictyostelia

Figure A1. *Dictyostelium lacteum* – group 3

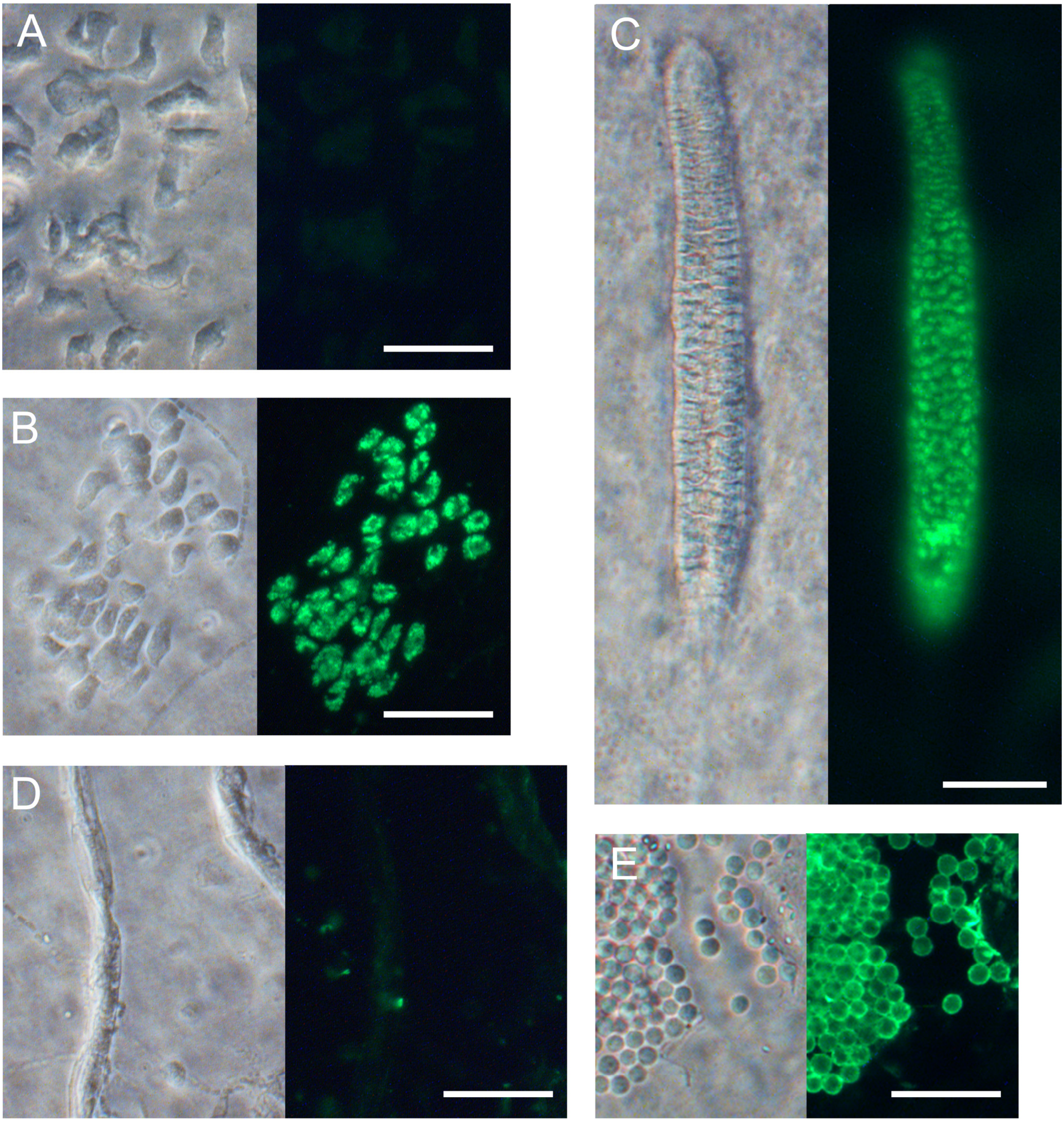

Phase contrast and fluorescent images of *D.lacteum* preaggregative cells (A), dissociated sorogens (B), intact sorogen (C), fruiting body stalks (D) and spores (E) stained with 1:2000 diluted antispore antibodies, raised in rabbit against a mixture of *D.discoideum* and *P.pallidum* spores, and poststained with FITC-conjugated donkey-anti-rabbit IgG. The images show that the spore antibodies specifically stain walls of the round spores of *D.lacteum* and vesicles in sorogens, that presynthesize the spore wall. Bar: 20 µm.

Figure A2. ***Polysphondylium pallidum*** **– group 2**

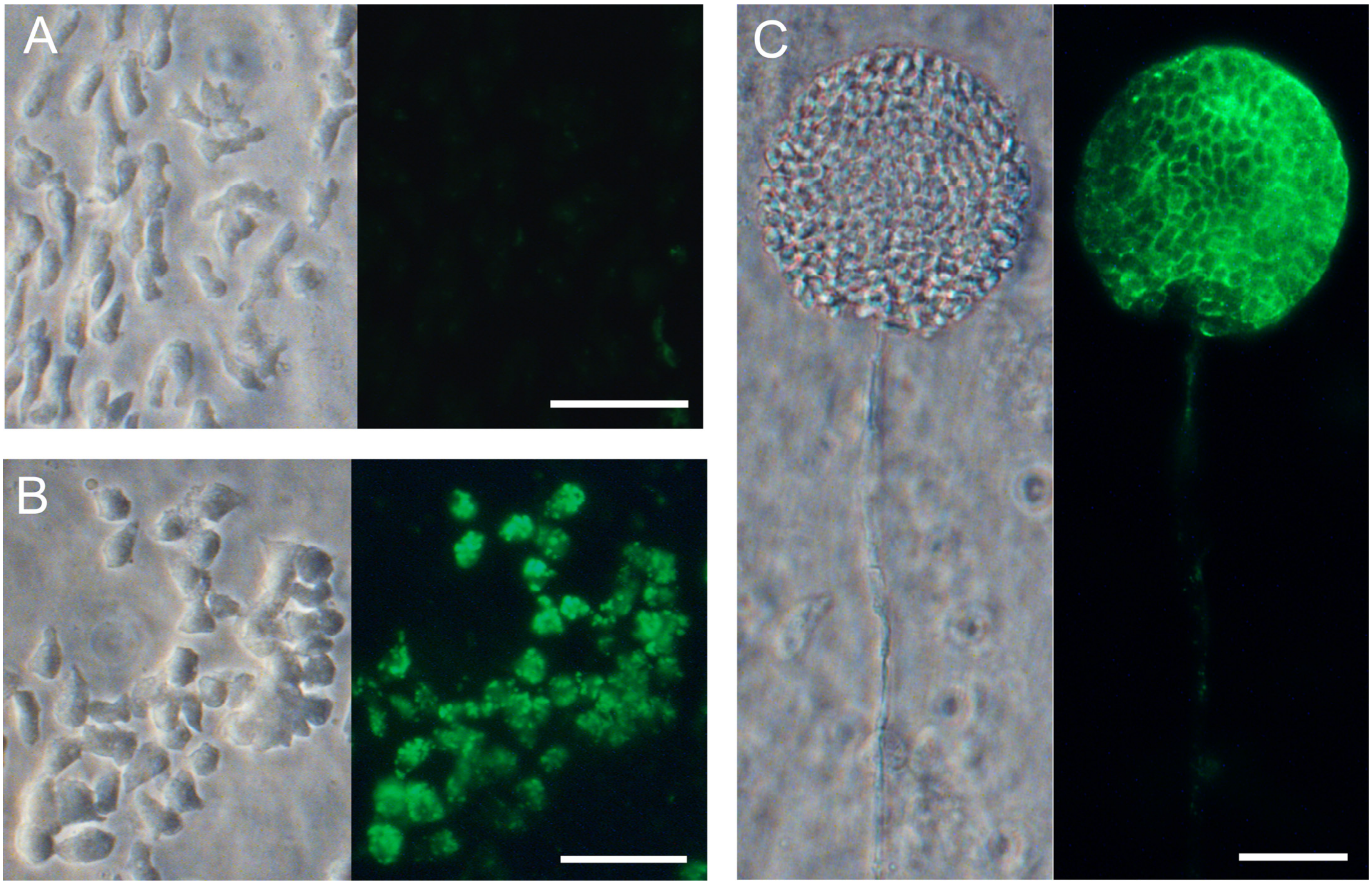

P. pallidum preaggregative cells (A), dissociated sorogens (B) and intact fruiting body (C) stained with antispore antibodies as described for figure A1. Bar: 20 µm.

Figure A3. Dictyostelium fasciculatum – group 1

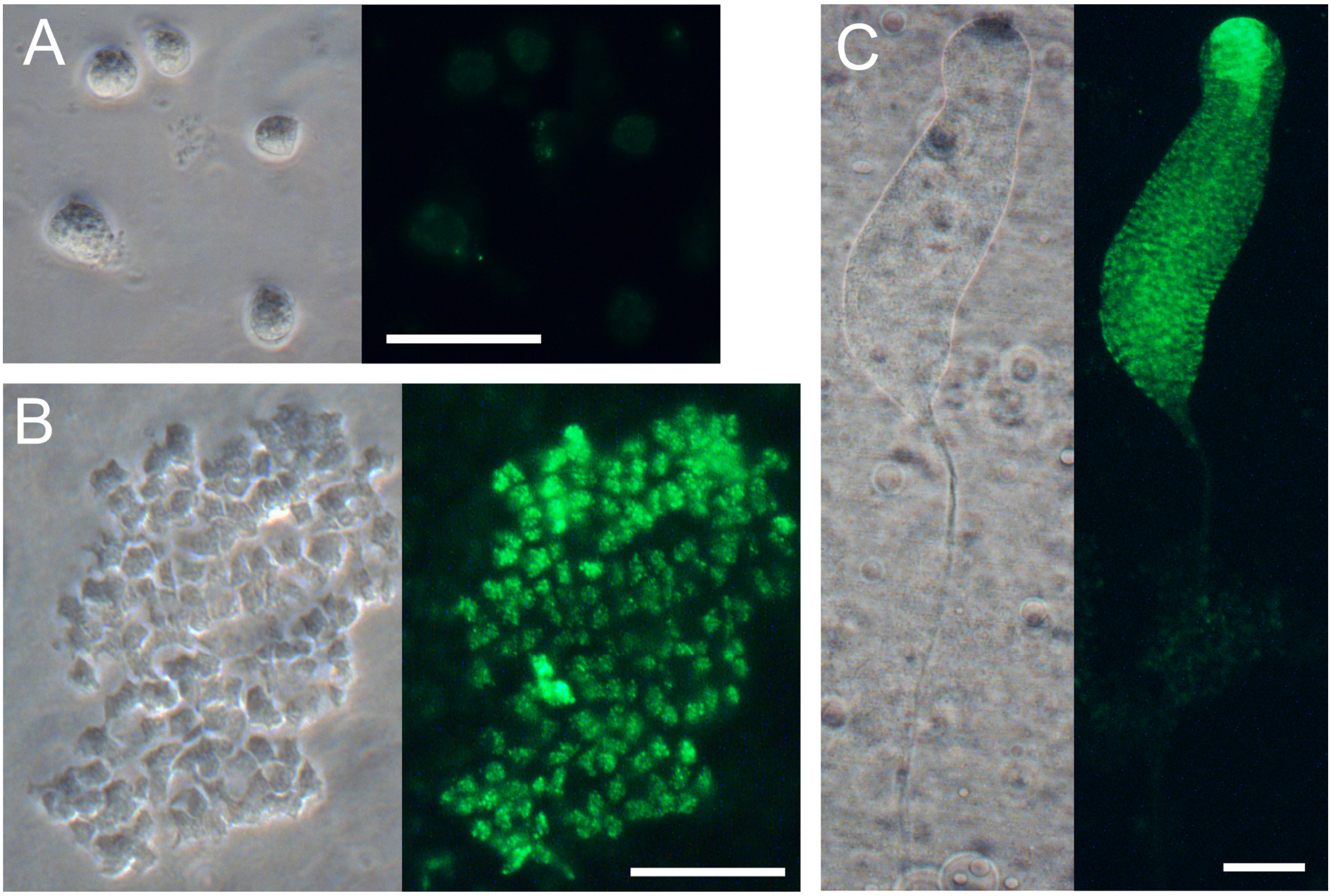

D. fasciculatum preaggregative cells (A), dissociated sorogens (B) and intact sorogen (C) stained with antispore antibodies as described for figure A1. Note that all cells from the dissociated sorogens contain stained granules. In *D.fasciculatum* and seven other species in group 1, the antispore antibodies also stain the walls of newly formed stalk cells (see also figure 2Db,c), but this staining is lost when the stalk matures. Bar: 20 µm.
